# Successful Treatment of Recalcitrant Pediatric Pemphigus Vulgaris With Rituximab

**DOI:** 10.1177/00099228221140801

**Published:** 2022-11-29

**Authors:** Marely Santiago-Vázquez, Valeria J. González-Molina, Frances M. Rodriguez-Ramos, Francisco Colón-Fontanez, Osward Y. Carrasquillo

**Affiliations:** 1 Department of Dermatology, University of Puerto Rico School of Medicine, San Juan, PR, USA; 2 Dermatology Residency Program, HCA Corpus Christi Medical Center–Bay Area, McAllen, TX, USA; 3 Department of Pediatrics, University of Miami Health System, Miami, FL, USA; 4 Department of Dermatology, University of North Carolina School of Medicine, Chapel Hill, NC, USA

## Introduction

Pemphigus vulgaris (PV) affecting the pediatric population is extremely rare, representing less than 3% of all PV cases.^1^ Conventional treatment with systemic steroids with or without adjuvant immunosuppressants used in adult cases of PV is often employed. However, pediatric cases often present a therapeutic challenge given their increased susceptibility to systemic therapy’s associated risks. To date, no specific guidelines regarding treatment strategies in this patient population exist. We describe a 14-year-old Hispanic female patient with severe, recalcitrant PV with suboptimal treatment response to systemic steroids and intravenous immunoglobulin (IVIG) successfully treated with rituximab (RTX), showing control of disease in as early as 2 weeks after treatment initiation. To our knowledge, only 45 cases of pediatric PV successfully treated with RTX have been reported in the literature, highlighting its use as a safe therapeutic option in this patient population.^1-17^

## Case Report

**Figure 1.**
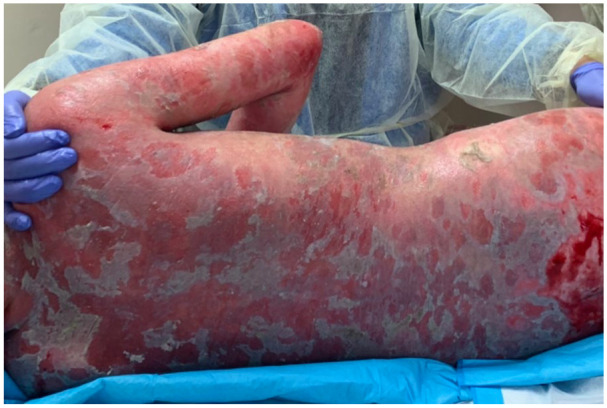
Note multiple erosions and scattered flaccid bullae involving almost 100% of patient’s back.

**Figure 2.**
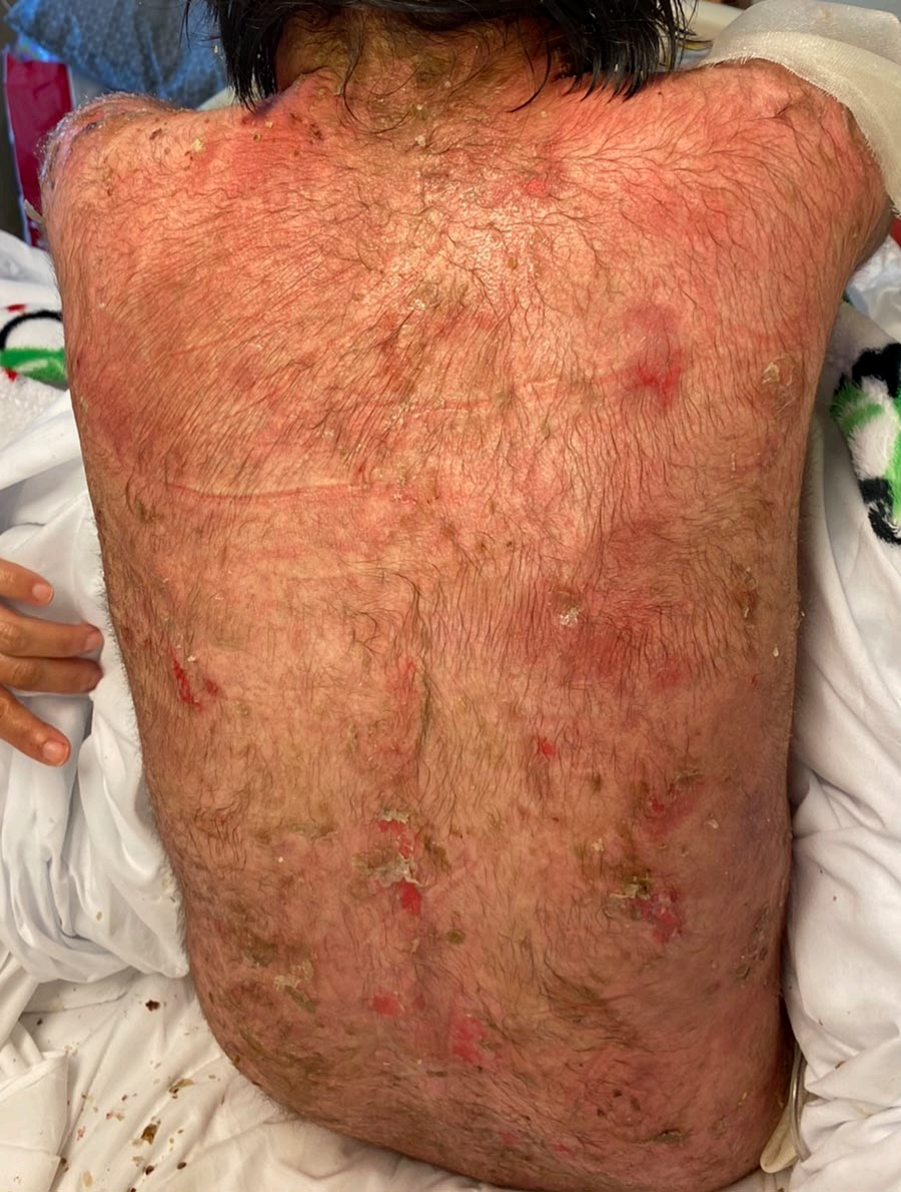
Significant re-epithelialization 4 weeks after the fourth cycle of rituximab. Note few scattered erosions and crusted plaques.

A 14-year-old Hispanic female patient presented to our clinics for evaluation of 1-month history of multiple scattered flaccid vesicles and erosive lesions involving the trunk, extremities, and oral mucosa. Lesional and perilesional skin biopsy was obtained, confirming the diagnosis of PV. At this time, treatment with systemic oral corticosteroids (0.5 mg/kg/d) was initiated with significant improvement. However, within 1 month of therapy, the patient had a severe relapse of disease complicated by sepsis requiring hospitalization (Figure 1). Treatment with intravenous (IV) antibiotics and IV steroids (methylprednisolone 40 mg) was initiated. After control of disease was achieved approximately 2 weeks following IV steroids, the patient was transitioned to oral prednisone 60 mg and mycophenolate mofetil 500 mg daily. Given suboptimal treatment response and progressive disease, IVIG treatment (15 g/d [0.4 mg/kg/d]) for 5 days was administered. Despite the previous treatment regimen, the patient showed minimal improvement. In addition, the patient developed side effects from the therapy, including recurrent infections, Cushingoid features, hypertrichosis, and edema. To minimize ongoing prolonged steroid use, treatment with RTX (4 cycles of 375 mg/m^2^, 1 week apart) was initiated. Baseline laboratories prior to initial RTX cycle revealed elevated markers for CD19 and CD20 B cells (25.8%, 25.8%), decreased markers for CD4 T cells (33.4%) and elevated serum anti-desmoglein (dsg) 1 and 3 antibodies (189 U/mL and 122 U/mL, respectively). Our patient tolerated RTX therapy well with no adverse effects showing rapid clinical remission (no new lesions) in as early as 2 weeks after initial infusion (Figure 2). Following RTX therapy, our patient continued with sustained clinical remission while on low dose steroids. Following 6 months after RTX infusion, systemic steroids were successfully discontinued. At the last 18-month follow-up, our patient has remained disease free off all therapy, with serial serum anti-dsg 1/3 levels persisting below normal levels and no relapse episodes.

## Discussion

Rituximab is a chimeric monoclonal antibody that targets the CD20 antigen present on the cell surface of B cells.^18^ Despite its approval in adults with new-onset moderate to severe PV or those with recalcitrant disease, data regarding its use in pediatric cases of PV currently remain off-label. Rituximab appears to show promising results for the management of pediatric PV.^19^ In 2019, Bilgic et al reviewed the literature describing a total of 30 cases of pediatric pemphigus (25 PV; 5 pemphigus foliaceous) successfully treated with RTX.^3^ Since then, 21 more pediatric PV cases, including ours, have been described, resulting in 46 patients ^1-17^ (Table 1). At diagnosis and at treatment initiation, mean age was 12.7 (range = 1.5-17) and 13.9 (range = 4.5-17) years, respectively. Following RTX therapy, patients were followed up for a mean period of 30 (range = 5-103) months. The most frequently used RTX dosing regimen in pediatric PV cases was 4 weekly infusions of 500 mg (n = 16), followed by 2 cycles of 500 mg infusions, 15 days apart (n = 9) and 4 weekly infusions of 375 mg/m^2^ body surface area (BSA) (n = 7), respectively.

**Table 1.** Summary of Pediatric Cases of Pemphigus Vulgaris Treated With Rituximab.

Total of patients, no.	46
Gender, no	21 F, 25 M
Mean age at diagnosis, years (range)	12.7 (1.5-17)
Mean age at initiation of RTX tx, years (range)	13.9 (4.5-17)
Mean pre-dsg 1/3 levels, U/mL	453.5, 415.2
RTX dosing regimen	
Body surface area dosing regimen	
500 mg weekly × 4 wks	17
375 mg/m^2^ BSA weekly × 4 wks	7
Fixed dosing regimen	
500 mg ×2, 2 wks apart	8
100 mg × 2, 2 wks apart	4
375 mg/m^2^ BSA × 2, 2 wks apart	6
375 mg/m^2^ BSA × 2, 30 days apart	1
300 mg/m^2^ BSA × 2, 2 wks apart	1
350 mg/m^2^ BSA × 2, 2 wks apart	2
Mean total follow-up, months (range)	30 (5-103)
Clinical outcome at last follow-up, no	
CR off T	21
CR on T	12
PR off T	4
PR on T	3
CR	3
PR	1
Not mentioned	1
Death	1
Reported AE, no	24
Infusion reaction (fever, chills, dyspnea, tachychardia, urticaria)	10
Angioedema	3
HSV	1
Infection (not specific, URI)	2
Sepsis	2
Relapse episodes, no	12
Mean post-dsg 1/3 levels, U/mL	30.4, 101.9

Abbreviations: AE, adverse effects; BSA, body surface area; F, female; M, male; Tx, treatment; RTX, rituximab; dsg, desmoglein; wks, weeks; CR off T, complete remission off therapy; CR on T, complete remission on therapy; PR off T, partial remission off therapy; PR on T, partial remission on therapy; CR, clinical remission; PR, partial remission; HSV, herpes simplex virus; URI, upper respiratory infection; no, number.

Most cases (78%, n = 36) result in clinical remission following RTX therapy, with clinical outcome at last follow-up varying from complete remission (CR) requiring ongoing systemic immunosuppression (n = 12) to CR off all therapy (n = 21). In 2 cases, CR was reported as treatment outcome, but details regarding ongoing therapies were unclear.^4,12^ Of all pediatric patients treated with RTX therapy, approximately 26% (n = 12) report a relapse of disease, the majority occurring within a mean period of 13 (range = 8-20) months after initial RTX therapy. Adverse effects associated with RTX were reported in approximately 35% of patients (n = 16). Of these, infusion reactions were the most commonly reported (mild infusion reactions, n = 12; angioedema, n = 3), followed by infections (n = 5). A summary of the reported cases, including clinical characteristics, treatment regimen, and outcome, is depicted in Table 2. Although pediatric patients treated with RTX are thought to have an increased risk of developing severe infections, sepsis was reported in only 2 cases, one of which resulted in death.^8,12^ At 12 months after RTX therapy, our patient has achieved CR off all treatment with no reported adverse effects or relapse episodes. Whether or not patients benefit from additional maintenance RTX infusions during follow-up to ensure sustained clinical remission is still a subject of great debate.^3,4^ In this review, a total of 18 patients received additional RTX infusions. In our case, at 6- and 12-month follow-up evaluations, our patient showed sustained clinical response; thus, additional maintenance infusions were deferred. Also, follow-up serum anti-dsg 1 and 3 antibody levels remained below normal limits and CD19 B-cell markers remained <1%, supporting sustained clinical response.

**Table 2.** Pediatric Cases of Pemphigus Vulgaris Treated With Rituximab.

Ref.	Case no	Sex/age at onset of PV (yrs)	Previous TX	AE due to previous TX	Age at initiation of RTX (yrs)	Indications for RTX/dosing regimen	Adjuvant TX	Additional RTX doses	AE due to RTX	Clinical outcome at last F/U visit	Total F/U (months)	Relapse	Serum dsg1/3 levels (pre/post RTX)	
Our case	1	F/13	CS, TS, MMF, IVIG	Sepsis, CUSH. hirsutism, edema	14	RD 375 mg/m^2^ weekly for 4 wks	TS, PSL (5-60 mg/d)	N	N	CR off T	6	No	189 U/mL and 122 U/mL	25 U/mL and 17 U/mL
Kianfar et al^1^	2	M/14	CS, AZT, MMF, ILCS	NR	17	RD 500 mg weekly for 4 wks	PSL (0.25 mg/kg/d)	1 cycle (4 infusions)	N	PR off T	103	Yes	NM	NM
	3	F/14	CS, AZT	CUSH, hirsutism acne, striae, osteopenia, ↑LFT	17	AE to previous tx/ 500 mg weekly for 4 wks	PSL (0.1 mg/kg/d)	N	Chills and fever	CR on T	18	No	NM	NM
	4	F/14	None	NR	14	Severe/500 mg weekly for 4 wks	PSL (1 mg/kg/d)	N	N	CR on T	28.5	Yes	NM	NM
	5	F/16	None	NR	16	Severe/500 mg weekly for 4 wks	PSL (1.8 mg/kg/d)	N	Fever	CR off T	58	No	NM	NM
	6	M/17	None	Dyspepsia	17	Severe, intolerance to PSL/500 mg weekly for 4 wks	PSL (0.8 mg/kg/d)	1 cycle (4 infusions)	Dyspnea	PR on T	57	Yes	NM	NM
	7	F/16	CS, AZT	NR	17	RD/500 mg weekly for 4 wks	PSL (0.5 mg/kg/d)	1 cycle (4 infusions)	Dyspnea, rigor, tachycardia	PR off T	51	Yes	NM	NM
	8	F/16	CS, AZT, MMF	↑LFT	17	RD/500 mg weekly for 4 wks	PSL (1.1 mg/kg/d)	N	N	CR on T	102	Yes	NM	NM
	9	F/17	None	NR	17	Severe/500 mg weekly for 4 wks	PSL (1.1 mg/kg/d)	2 infusions	N	PR on T	51.5	Yes	NM	NM
	10	F/16	CS	NR	17	RD/500 mg weekly, for example, 4 wks	PSL (0.9 mg/kg/d)	N	Dyspnea	CR on T	5	No	NM	NM
	11	M/11	CS, AZT	NR	11	RD/500 mg weekly for 2 wks	PSL (2 mg/kg/d)	2 infusions	Fever, infection	CR off T	97	Yes	30 U/mL and 137 U/mL	NM
Broshtilova et al^2^	12	F/14	CS, DAP	CUSH, delayed growth, skin atrophy, striae, hirsutism, menstrual irregularity, infections	14	RD and due to AEs of previous therapy/2 dose of 375 mg/m^2^ 30 days apart	NM	NM	N	CR	34	No	270 U/mL and 314 U/mL	255 U/mL and 251 U/mL (first infusion) 202 U/mL and 308 U/mL (second infusion)
Bilgic-Temel et al^3^	13	M/16	CS, AZA	Psychosis, OP, wt gain	17	RD and severe adverse events/2 dose 1000 mg 15 days apart	MP (0.5-1 mg/kg/d)	3 cycles of fixed dose (500 mg)	N	CR off T	60	No	NM	NM
	14	M/14	CS, AZA, DAP, MMP, IVIG	Cataract, lymphopenia	17	RD and adverse events/2 doses 1000 mg 15 days apart	IVIG	N	N	PR off T	49	No	NM	NM
	15	M/13	CS, AZA, DAP	NR	17	RD/2 dose 1000 mg 15 days apart	DAP	N	N	PR off T	60	No	NM	NM
	16	F/9	CS, AZA, IVIG, DAP	OP, anemia	13	RD and severe/2 dose 500 mg 15 days apart	MP, AZA	3 cycles of fixed dose (500 mg)	N	CR off T	25	Yes	NM	NM
	17	F/10	CS, IVIG, ILS, TS	OP, wt gain, CUSH	11	RD and severe adverse events/375 mg/m^2^ weekly for 4 weeks	MP, ILS, TS	3 cycles (2 doses of RTX of 375 mg/m^2^ weekly for 4 wks and 1 prophylactic dose of RTX of 375 mg/m^2^ weekly for 2 weeks)	N	CR off T	19	Yes	NM	NM
Gupta et al^4^	18	M/12	CS, AZA	NR	12	RD, contraindication to steroids, severe/2 doses of 500 mg 2 wks apart	CS (5-20 mg/d)	N	Infusion reaction (1) HZV (1)	CR off T	12	No	31.8 U/mL and 17.1 U/mL	11.3 U/mL and 3.1 U/mL
	19	F/9	CS, AZA	Anemia	9		CS (5-20 mg/d)	N		CR off T	12	No	357 U/mL and 219 U/mL	98 U/mL and 77 U/mL
	20	F/11	CS, CP	NR	11		CP (50 mg/d), CS (5-20 mg/d)	N		CR on T	12	No	99.1 U/mL and 103.4 U/mL	3.4 U/mL and 6.7 U/mL
	21	M/12	CS, CP	NR	12		CP (50 mg/d), CS (5-20 mg/d)	N		CR on T	12	No	184.6 U/mL and 94 U/mL	80.5 U/mL and 64 U/mL
	22	M/9	CS, AZA	NR	9		CS (5-20 mg/d)	N		CR off T	12	No	150 U/mL and 10.8 U/mL	47 U/mL and 1.8 U/mL
Salman et al^5^	23	M/14	CS, AZA, IVIG, DAP	Osteopenia, CUSH, ↑LFT	14	RD/2 doses of 375 mg/m^2^ 15 days apart	Low dose CS (0.1-0.2 mg/kg/d)	3 cycles	N	CR on T	24	No	NM	NM
	24	M/16	CS, AZA, IVIG, MMF	Cataracts, osteopenia, lymphopenia	16	RD/2 doses of 375 mg/m^2^ 15 days apart	Low dose CS (0.1-0.2 mg/kg/d)	1 cycles	N	CR off T	44	No	NM	NM
Buch JY et al^6^	25	F/11	CS, AZA	Hyperglycemia, CUSH	11	RD/2 doses of 300 mg/m^2^ 15 days apart	40 mg PSL	1 cycle	N	CR on T	12	1 patient relapsed (not specific)	20.2 U/mL and 143.4 U/mL	1.8 U/mL and 5.7 U/mL
	26	M/11	CS, AZA	CUSH, wt gain, HTN	12	RD/2 doses of 300 mg/m^2^ 15 days apart	40 mg PSL	N	N	CR on T	12		237 U/mL and 194.6 U/mL	64 U/mL and 94 U/mL
	27	F/9	CS	CUSH, wt gain	9	RD/2 doses of 300 mg/m^2^ 15 days apart	40 mg PSL	1 cycle	N	CR on T	12		228 U/mL and 229 U/mL	47.3 U/mL and 2.5 U/mL
Kincaid et al^7^	28	F/4	CS, IVIG, AZA	HTN, CUSH, delayed growth	4 and 5	RD/2 doses of 375 mg/m^2^ 15 days apart	CS, AZA	2 cycles	Mild infusion reaction, urticaria, low-grade fever	CR off T	24	Yes	320 U/mL	NM
Kong et al^8^	29	M/9	CS, MTX, MMF, Cyp, DAP	NR	NM	RD/2 doses of 750 mg/m^2^ 2 wks apart	MTX, CS, MMF	1 cycle	Neutropenia and sepsis	CR on T	25	No	NM	NM
Vinay et al^9^	30	M/NS	CS, AZA, DAP	NR	9	RD and severe/2 doses of 375 mg/m^2^ 15 days apart	CS (0.5-1 mg/kg/d)	N	Angioedema	CR off T	36	No	1372.80 U/mL and 888.80 U/mL	0.5 U/mL and 14.22 U/mL
	31	M/NS	CS, AZA, DAP	NR	11	Severe/2 doses of 375 mg/m^2^ 15 days apart	CS (0.5-1 mg/kg/d)	N	Infusion reaction	CR off T	8	No	248.52 U/mL and 575.20 U/mL	Lost to F/U
	32	M/NS	CS, AZA, DAP, MMF	NR	17	RD and severe/2 doses of 500 mg/m^2^ 15 days apart	AZA (2 mg/kg/d), CS (0.5-1 mg/kg/d)	N	N	CR off T	19	No	67.30 U/mL and 130.33 U/mL	7.56 U/mL and 4.48 U/mL
	33	M/NS	CS	CUSH	17	RD and SEs of previous therapy/2 doses of 500 mg/m^2^ 15 days apart	AZA (2 mg/kg/d), CS (0.5-1 mg/kg/d)	1 cycle	Infusion reaction	CR on T	18	No	1148.46 U/mL and 116.09 U/mL	94.04 U/mL and 14.79 U/mL
	34	F/NS	CS	NR	17	Severe/2 doses of 500 mg/m^2^ 15 days apart	CS (0.5-1 mg/kg/d)	N	N	CR off T	17	No	1743.74 U/mL and 87.43 U/mL	18.10 U/mL and 0.86 U/mL
	35	F/NS	CS, AZA	NR	13	RD/2 doses of 500 mg/m^2^ 15 days apart	CS (0.5-1 mg/kg/d)	N	Upper respiratory tract infection	CR off T	14	No	165.11 U/mL and 340.25 U/mL	1.83 U/mL and 6.26 U/mL
	36	M/NS	CS, AZA	NR	12	RD/2 doses of 500 mg/m^2^ 15 days apart	CS (0.5-1 mg/kg/d)	1 cycle	Angioedema	CR off T	12	No	107.11 U/mL and 134.81 U/mL	2.98 U/mL and 274.17 U/mL
Didona et al^10^	37	M/11	CS, IVIG	Wt gain, CUSH.	11	RD/2 doses of 375 mg/m^2^ 18 days apart	NM	N	N	CR	10	No	NM	NM
	38	M/17	DP, AZA, MMF	NR	NM	RD/2 doses of 500 mg/m^2^ 15 days apart	CS	N	N	NM	12	NM	NM	NM
Chen et al^11^	39	M/17	CS, IVIG	NR	17	RD/500 mg/m^2^ weekly for 4 wks	CS	N	N	CR off T	NS	NM	NM	NM
Kanwar et al^12^	40	M/9	CS, DP, AZA	NR	NM	RD and severe/2 doses of 375 mg/m^2^ 15 days apart	PSL (1-1.5 mg/kg/d)	N	Angioedema	CR off T	11.5	NM	1372.80 U/mL and 888.80 U/mL	0.12 U/mL and 742.40 U/mL
	41	M/17	CS, CP	NR	NM	RD and severe/2 doses of 1000 mg/m^2^ 15 days apart	PSL (1-1.5 mg/kg/d)	N	Sepsis	Death	N/A	NM	95.73 U/mL and 25.60 U/mL	NA
Reguai et al^13^	42	F/14	CS, IVIG	Mood changes, acne, gastric ulcer	14	Severe AEs on previous therapy/375 mg/m^2^ weekly for 4 wks	NM	2 cycles	N	CR off T	62	Yes	65 U/mL and 160 U/mL	2 U/mL and 44 U/m:
Fuertes et al^14^	43	M/1.5	CS, AZA, DAP, Gold	CUSH	14	RD/375 mg/m^2^ weekly for 4 wks	CS (prednisone 20 mg/d slowly tapered)	N	N	CR	18	No	157 U/mL and 3 U/mL	10 U/mL and 1 U/mL
Schmidt et al^15^	44	F/17	CS, IVIG, MTX, AZA, MMF,	NR	NM	RD/375 mg/m^2^ weekly for 4 wks	MMF and MP	N	N	CR off T	7	NM	NM	NM
Schmidt et al^16^	45	M/11.5	CS, AZA, DAP, MMF, CP, IVIG, MMF	Osteopenia, infections, CUSH, growth delay	14	RD/375 mg/m^2^ weekly for 4 wks	MMF (1-5 g/d), MP (12 mg/d), IVIG (10 g after 1st and 4th infusion)	N	N	CR off T	24	No	NM	NM
Kong et al^17^	46	F/10	CS, AZA, MMF, IVIG, plasmapheresis	Myelosuppression, infections	17	RD/375 mg/m^2^ weekly for 4 wks, then every 4 or 8 wks	CS	Y, 375 mg/m^2^/wk × 4 every 4-8 weeks	N	CR on T	17	NM	2079 U/mL and 4616 U/mL	33 U/mL and 564 U/mL

Abbreviations: AE, adverse effects; AZA, azathioprine; CS, systemic corticosteroids; CP, cyclophosphamide; Cys, cyclosporine; CR off T, complete remission off therapy; CR on T, complete remission on therapy; CR, complete remission; CUSH, cushingoid; DAP, dapsone; Dsg, desmoglein; F/U, follow-up; IVIG, intravenous immunoglobulin; ILCS, intralesional steroids; LFT, liver function tests; MMF, mycophenolate mofetil; MTX, methotrexate; MP, methylprednisolone; NR, not reported; NM, not mentioned; OP, osteoporosis; PR on T, partial remission on therapy; PSL, prednisolone; Ref, references; RTX, rituximab; RD, refractory disease to conventional therapy; TS, topical steroids; wt, weight; wks, weeks.

## Conclusion

We describe a 14-year-old Hispanic female patient who achieved sustained CR off treatment with no adverse effects or relapses reported at 18-month follow-up evaluation after successfully being treated with RTX. To our knowledge, CR off therapy has also been reported in 22 other cases. Given the vulnerability of this patient population, there is an increasing need for new therapeutic options. Our case is relevant because it supports the growing number of cases reporting successful treatment for pediatric PV using RTX. Physicians treating pediatric PV should be aware of the use of RTX as a therapeutic option in cases of recalcitrant pediatric PV, achieving prompt control of the disease. However, the need for randomized clinical trials to assess the role and efficacy of RTX on pediatric patients with PV remains.

## Author Contributions

OCY and FC conceptualized and proposed the case report, edited the report, and provided guidance throughout. OYC, MS, VJG and FMR completed the literature review, wrote the first draft of the manuscript, and edited the report. MS, VJG, and FMR created the tables. All authors approved the final manuscript as submitted and agree to be accountable for all aspects of the work.

## References

[1] Kianfar N Dasdar S Mahmoudi H Tavakolpour S Balighi K Daneshpazhooh M . Rituximab in childhood and juvenile autoimmune bullous diseases as first-line and second-line treatment: a case series of 13 patients. J Dermatolog Treat. 2020;33:869-874. 32589481 10.1080/09546634.2020.1788702

[2] Broshtilova V Vassileva S . A case of pediatric pemphigus treated with rituximab—our experience. Asian J Res Dermatol Sci. 2019;2(1):1-6.

[3] Bilgic-Temel A Özgen Z Harman M Kapıcıoğlu Y Uzun S . Rituximab therapy in pediatric pemphigus patients: a retrospective analysis of five Turkish patients and review of the literature. Pediatr Dermatol. 2019;36(5):646-650. 31355479 10.1111/pde.13926

[4] Gupta J Raval RC Shah AN , et al. Low-dose rituximab as an adjuvant therapy in pemphigus. Indian J Dermatol Venereol Leprol. 2017;83(3):317-325. 28366912 10.4103/ijdvl.IJDVL_1078_14

[5] Salman A Tekin B Yucelten D . Autoimmune bullous disease in childhood. Indian J Dermatol. 2017;62(4):440. 10.4103/ijd.IJD_366_16 PMC5527737 28794567

[6] Buch JY Raval RC . Successful treatment of refractory childhood vesiculobullous disorders with rituximab: a study of five cases. Indian J Paediatr Dermatol. 2016;17:104-107.

[7] Kincaid L Weinstein M . Rituximab therapy for childhood pemphigus vulgaris. Pediatr Dermatol. 2016;33(2):e61-e64. 10.1111/pde.12744 26765543

[8] Kong YL Lim YL Chandran NS . Retrospective study on autoimmune blistering disease in paediatric patients. Pediatr Dermatol. 2015;32(6):845-852. 26391853 10.1111/pde.12684

[9] Vinay K Kanwar AJ Sawatkar GU Dogra S Ishii N Hashimoto T . Successful use of rituximab in the treatment of childhood and juvenile pemphigus. J Am Acad Dermatol. 2014;71(4):669-675. 25022850 10.1016/j.jaad.2014.05.071

[10] Didona D Paolino G Donati M Caposeina D Calvieri S Didona B . Resolution of a case of pediatric pemphigus vulgaris treated with rituximab. Acta Dermatovenerol Croat. 2014;22(4):288-290. 25580789

[11] Chen IH Mu SC Tsai D Chou YY Wang LF Wang LJ . Oral ulcers as an initial presentation of juvenile pemphigus: a case report. Pediatr Neonatol. 2013;57(4):338-342. 24295781 10.1016/j.pedneo.2013.08.008

[12] Kanwar AJ Tsuruta D Vinay K , et al. Efficacy and safety of rituximab treatment in Indian pemphigus patients. J Eur Acad Dermatol Venereol. 2013;27(1):e17-e23. 10.1111/j.1468-3083.2011.04391.x 22176540

[13] Reguiai Z Tabary T Maizieres M Bernard P . Rituximab treatment of severe pemphigus: long-term results including immunologic follow up. J Am Acad Dermatol. 2012;67:623-629. 22261417 10.1016/j.jaad.2011.12.019

[14] Fuertes I Guilabert A Mascaró JM Jr Iranzo P . Rituximab in childhood pemphigus vulgaris: a long-term follow-up case and review of the literature. Dermatology. 2010;221(1):13-16. 20389028 10.1159/000287254

[15] Schmidt E Seitz CS Benoit S Bröcker EB Goebeler M . Rituximab in autoimmune bullous diseases: mixed responses and adverse effects. Br J Dermatol. 2007;156(2):352-356. 17223877 10.1111/j.1365-2133.2006.07646.x

[16] Schmidt E Herzog S Bröcker EB Zillikens D Goebeler M . Long-standing remission of recalcitrant juvenile pemphigus vulgaris after adjuvant therapy with rituximab. Br J Dermatol. 2005;153(2):449-451. 16086770 10.1111/j.1365-2133.2005.06740.x

[17] Kong HH Prose NS Ware RE Hall RP III . Successful treatment of refractory childhood pemphigus vulgaris with anti-CD20 monoclonal antibody (rituximab). Pediatr Dermatol. 2005;22(5):461-464. 16191003 10.1111/j.1525-1470.2005.00118.x

[18] Eming R Nagel A Wolff-Franke S Podstawa E Debus D Hertl M . Rituximab exerts a dual effect in pemphigus vulgaris. J Invest Dermatol. 2008;128(12):2850-2858. 18563178 10.1038/jid.2008.172

[19] Joly P Maho-Vaillant M Prost-Squarcioni C , et al. First-line rituximab combined with short-term prednisone versus prednisone alone for the treatment of pemphigus (Ritux 3): a prospective, multicentre, parallel-group, open-label randomised trial. Lancet. 2017;389(10083):2031-2040. 28342637 10.1016/S0140-6736(17)30070-3

